# Anesthetic management of patients with carnitine deficiency or a defect of the fatty acid β-oxidation pathway: A narrative review

**DOI:** 10.1097/MD.0000000000028853

**Published:** 2022-02-18

**Authors:** Ho Kyung Yu, Seong-Ho Ok, Sunmin Kim, Ju-Tae Sohn

**Affiliations:** a Department of Anesthesiology and Pain Medicine, Gyeongsang National University Changwon Hospital, Changwon-si, Republic of Korea; b Department of Anesthesiology and Pain Medicine, Gyeongsang National University College of Medicine, 15 Jinju-daero 816 beon-gil, Jinju-si, Gyeongsangnam-do, Republic of Korea; c Institute of Health Sciences, Gyeongsang National University, Jinju-si, Republic of Korea; d Department of Anesthesiology and Pain Medicine, Gyeongsang National University College of Medicine, Gyeongsang National University Hospital, 15 Jinju-daero 816 beon-gil, Jinju-si, Gyeongsangnam-do, Republic of Korea.

**Keywords:** anesthesia, carnitine deficiency, carnitine shuttle, defects in the fatty acid β-oxidation pathway, fatty acid β-oxidation disorder, hypoglycemia

## Abstract

Carnitine is essential for the transport of long-chain fatty acids from the cytoplasm to the mitochondrial matrix. The carnitine shuttle transports long-chain fatty acylcarnitine to the mitochondrial matrix. Subsequently, long-chain fatty acyl CoA, which is split from long-chain fatty acylcarnitine by carnitine palmitoyltransferase II, undergoes fatty acid β-oxidation. Acetyl CoA is produced from long-chain fatty acyl CoA via fatty acid β-oxidation and aids in the synthesis of adenosine triphosphate via the tricarboxylic acid cycle and electron transport chain. In addition, in the fasting state, it leads to ketone body production in the liver and glucose production via gluconeogenesis. However, patients with compromised fatty acid β-oxidation, owing to carnitine deficiency as well as defects in carnitine transport and the fatty acid β-oxidation pathway, develop hypoglycemia, cardiomyopathy, arrhythmia, and hypotonia. These conditions are attributed to the accumulation of released fatty acids and acylcarnitine. This review aimed to shed light on the anesthetic management of patients with compromised fatty acid β-oxidation undergoing various surgeries by assessing relevant case reports associated with fatty acid β-oxidation disorder in PubMed. Pre-anesthetic and intraoperative evaluation should include monitoring of glucose and carnitine levels and specific cardiac tests, such as echocardiography. Considering that propofol is dissolved in 10% long-chain fatty acids, propofol infusion should be avoided because of increased long-chain fatty acid loading in patients with compromised fatty acid β-oxidation. Thus, anesthesia using opioids (remifentanil and fentanyl), midazolam, dexmedetomidine, etomidate, and non-depolarizing neuromuscular blocking agents would be appropriate in such patients.

## 1 Introduction

Carnitine (β-hydroxoy-γ-N-trimethylaminobutyric acid) is an essential water-soluble nutrient required for transporting long-chain fatty acids from the cytoplasm to the mitochondrial matrix.^[1]^ Dietary intake accounts for 75% of the total body carnitine in non-vegetarians, and the remaining 25% is endogenously produced from lysine and methionine in the liver and kidneys.^[1]^ Despite the difference in dietary carnitine intake between vegetarians and non-vegetarians, free plasma carnitine levels are constantly maintained within the reference limits (20–50 μM/L) because of renal resorption and excretion associated with an excess amount of carnitine.^[1]^ The skeletal muscle contains more than 95% of the total body carnitine, and the remaining carnitine is present in the liver, heart, and kidneys.^[1]^ Carnitine contributes to lipid metabolism and subsequent energy production via fatty acid β-oxidation in the heart, liver, and skeletal muscle.^[1]^ Fatty acid β-oxidation is a physiological response to the energy shortage state of tissues that is caused by fasting, vigorous exercise, and stressful conditions (febrile illness, infection, and surgery).^[2]^ In addition, fatty acid β-oxidation is associated with synthesis of adenosine triphosphate via the downstream tricarboxylic acid cycle and electron transport chain and meets 80% energy demand of the heart, liver, and skeletal muscle.^[2]^ Thus, fatty acid β-oxidation disorder, which occurs because of carnitine deficiency and defects in the carnitine shuttle and fatty acid β-oxidation pathway, results in the development of hypoglycemia, cardiomyopathy, hepatomegaly, and rhabdomyolysis.^[2]^ These disorders cause Reye-like syndrome, arrhythmia, cardiomyopathy, and muscle weakness in the fasting state during later infancy and childhood.^[3]^ Rhabdomyolysis and cardiomyopathy due to fatty acid β-oxidation disorder are common adolescent- or adult-onset symptoms.^[3]^ The wide range of clinical presentations and severity depends on the type or the management of the disease. In particular, poor control of fatty acid β-oxidation disorder causes severe damage to patients with hypoglycemia-induced brain damage.^[^1^,^^4^^]^ The stress response to surgery and disease causes a derangement of metabolic and physiological responses, ultimately inducing hypercatabolism and hypermetabolism.^[5]^ The ratio of primary carnitine deficiency is 1:142,000 in newborn screening tests; however, carnitine deficiency reportedly produces ventricular arrhythmia and hypotension induced by subtoxic doses of bupivacaine, thereby suggesting that the deficiency increases the susceptibility to bupivacaine-induced cardiac toxicity.^[^1^,^^6^^,^^7^^]^ In addition, increased cortisol, catecholamine, and glucagon secretion because of surgery-induced stress response, which contributes to improved survival after trauma and surgery, stimulates lipolysis and ketone body production through fatty acid β-oxidation.^[8]^ Thus, by assessing relevant previous reports,^[^1^,^^5^^–^8^]^ this review aimed to shed light on the importance of anesthetic management in patients with fatty acid β-oxidation disorder (carnitine deficiency and defects in the fatty acid β-oxidation pathway) who are undergoing various surgeries.

## 2 Methods

**Figure 1:**
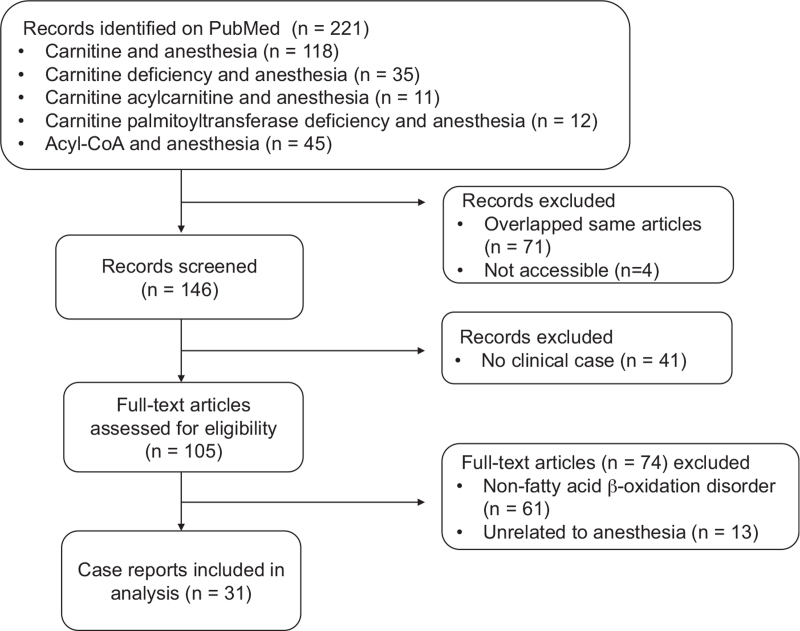
Flow chart for extracting relevant case reports from PubMed on anesthesia for patients with fatty acid β-oxidation disorder who underwent surgery. We used the following terms: “carnitine and anesthesia,” “carnitine deficiency and anesthesia,” “carnitine acylcarnitine and anesthesia,” “carnitine palmitoyltransferase deficiency and anesthesia,” and “acyl-CoA and anesthesia.”

We searched for relevant articles in PubMed (date: March 11, 2021 to August 15, 2021) using the following terms: “carnitine and anesthesia” (N = 118), “carnitine deficiency and anesthesia” (N = 35), “carnitine acylcarnitine and anesthesia” (N = 11), “carnitine palmitoyltransferase deficiency and anesthesia” (N = 12), and “acyl-CoA and anesthesia” (N = 45). A total of 221 articles were retrieved from the database (Fig. 1). We excluded overlapping (N = 71) and not accessible (N = 4, written in Japanese or Spanish, not accessible full text) articles (Fig. 1). The remaining 146 articles were screened, of which 41 non-clinical case reports were excluded (Fig. 1). We carefully reviewed the remaining articles (N = 105). Articles on non-fatty acid β-oxidation disorder (N = 61) and those unrelated to anesthesia (N = 13) were also excluded (Fig. 1). Eventually, we analyzed a total of 31 case reports (31 patients), with 1 case involving the same patient undergoing 2 surgeries (Fig. 1). Institutional review board approval is not necessary because this is an evidence-based narrative review.

### 2.1 Analysis of data

Pre-school (≤) and other (>6) age distributions were 10 and 21, respectively (Table 1). There were 14 men and 16 women (not recorded: N = 1) (Table 1). All patients (N = 31) displayed defects in the fatty acid β-oxidation pathway (N = 18; 58.6%), secondary carnitine deficiency (N = 5; 16.1%; valproic acid: N = 2; 3-methylcrotonyl-CoA carboxylase deficiency: N = 1; glutaryl-CoA dehydrogenase deficiency [glutaric aciduria type I]: N = 1; and isovaleryl CoA dehydrogenase deficiency [isovalemic acidemia]: N = 1), primary carnitine deficiency (N = 4; 12.9%), and defects in carnitine transport (N = 4, 12.9%) (Table 1). Defects in the fatty acid β-oxidation pathway (N = 18) included very long chain acyl-CoA dehydrogenase deficiency (N = 7), medium chain acyl-CoA dehydrogenase deficiency (N = 5), short chain acyl-CoA dehydrogenase deficiency (N = 3), and glutaric aciduria type II (N = 3) (Table 1). In addition, defects in carnitine transport (N = 4) involved carnitine palmitoyltransferase II (CPT-II) deficiency (N = 3) and carnitine palmitoyltransferase I (CPT-I) deficiency (N = 1) (Table 1). Twenty-eight patients (90.3%) were previously diagnosed with fatty acid β-oxidation disorder before anesthesia for various surgeries, including primary carnitine deficiency, defects in the fatty acid β-oxidation pathway, 3-methylcrotonyl-CoA carboxylase deficiency, isovaleryl CoA dehydrogenase deficiency, and CPT-II deficiency (Table 1). However, 3 patients (9.6%) were diagnosed with carnitine deficiency during admission (Table 1). In addition, the timing of diagnosis of fatty acid β-oxidation disorder was unavailable in 8 patients (Table 1). Some patients had pre-operative respiratory, cardiovascular, neurologic, and muscular symptoms because of fatty acid β-oxidation disorder, which included recurrent pneumonia, muscular pain, muscle weakness, rhabdomyolysis, seizure, heat burn, and lethargy (Table 1). Six patients (19.3%) had a family history of primary carnitine deficiency, CPT-I, and CPT-II deficiency, glutaric aciduria type II, medium-chain acyl-CoA dehydrogenase deficiency, and short-chain acyl-CoA dehydrogenase deficiency (N = 1 each) (Table 1). Seven patients (22.5%) pre-operatively received other medications, including anticonvulsants (valproic acid, clonazepam, clobazam, and levetiracetam), antianxiolytics (paroxetine), anticholinergics (benztropine), antipsychotics (ziprasidone), antidiabetics, and contraceptives (Table 1).

**Table 1:** Characteristics of patients with fatty acid β-oxidation disorder who underwent anesthesia for various surgeries.

			DFAO Hx.						Pre-anesthetic Mx. for DFAO		During anesthesia								After anesthesia
No.	Sex	Age	Type	T. of Dx.	Sx.	Tx.	FHx.	Other medication	Dextrose	Carnitine	LA	Route	GA	NMB		Other monitoring	Event	Tx for event	Outcome
														Agent	Monitor				
1^[9]^	M	8 mo	1CD	5 mo	MR	–	–	–	Yes	–	–	–	Vol	–	–	–	–	–	NS
2^[10]^	F	5	1CD	2 YA	Neu	Car	–	–	Yes	Yes	–	–	Vol	ND	–	–	–	–	NS
3^[11]^	M	13	1CD	This	MR	No	Yes	–	–	–	–	–	Vol	Dep	–	–	Severe	CPR	Died 1 year after rhabdomyolysis
4^[6]^	F	16	2CD (IA)	8 YA	GI	Car	–	NS	–	Yes	BPV	Inf	Vol	ND	–	Basic, CO_2_	Severe	Adrenaline	Mild left ventricle dysfunction
5^[12]^	M	57	1CD	15 YA	MR	Car	–	–	Yes	Yes	MPV	NA	TIVA (p)	ND	Yes	Basic, CO_2_, Glu, BT	–	–	NS
6^[13]^	F	30	2CD (MCCD)	28 YA	No	Car	–	NS	–	Yes	–	–	Vol (p)	–	–	Basic	–	–	NS
7^[14]^	F	31	DCT (CPT-II)	12 YA	MR	Diet	–	–	Yes	–	LA	NA	No	No	No	Basic, BT	NS	–	NS
8^[7]^	M	6	2CD (VPA)	This	No	No	–	Anticon	–	–	BPV	NA	Vol (p)	ND	–	Basic, CO_2_	Severe	CPR, LE adrenaline	Death, cerebral ischemic injury
9^[15]^	F	30	DCT (CPT-II)	22 YA	MR	Diet	Yes	–	Yes	–	Lido,BPV	NA	No	No	No	–	NS	–	NS
10^[16]^	F	28	DCT (CPT-II)	2 YA	Neu	Diet	–	–	Yes	Yes	RPV	NA	No	No	No	–	NS	–	NS
11^[17]^	F	3.5	DCT (CPT-I)	13 Mo	Neu, GI	–	Yes	–	–	No	No	No	Vol	Dep	–	–	Severe	Car, ICU care	Full recovery from coma
12^[18]^	F	23	2CD (GAT1)	Uk	Ren	Diet, Car	–	–	Yes	Yes	BPV	NA	No	No	No	–	NS	–	NS
13^[19]^	F	1.9	SCAD	Birth	–	–	Yes	–	Yes	Yes	–	–	Vol	ND	Yes	Basic, CO_2_, BT, Glu	–	–	NS
14^[20]^	M	47	2CD (VPA)	This	–	No	–	Anticon	–	–	BPV	Inf	TIVA (p)	ND	–	Basic, CO_2_ ABGA	NS	Car	NS
15^[21]^	M	12	SCAD	Uk	MR	–	–	–	Yes	–	–	–	Vol, IVA	ND	–	Basic	–	–	NS
16^[22]^	M	1.6	VLCAD	Birth	MR	MCT	–	–	Yes	–	–	–	Vol	ND	–	Basic, Glu	–	–	NS
17^[23]^	–	4	MCAD	1 YA	Neu	–	–	–	Yes	–	LA	Inf	–	–	–	–, Glu	–	–	NS
18^[24]^	F	11	GAT2	7 Mo	Neu, MR	Diet, Car	Yes	Anxio	Yes	–	–	–	TIVA	ND	Yes	Basic, BT, Glu	–	–	NS
19^[25]^	M	3	VLCAD	Uk	MR	–	–	–	Yes	–	–	–	Vol, IVA	–	–	–	–	–	NS
20^[26]^	M	9	MCAD	Birth	BR	Diet	–	–	No	No	–	–	Vol	No	No	Basic, Glu	–	–	NS
21^[27]^	F	28	VLCAD	Uk	MR	Car	–	–	Yes	Yes	–	–	Vol	ND	–	–	–	–	NS
22^[28]^	M	2	MCAD	Uk	No	Car	–	–	Yes	Yes	–	–	Vol (p)	–	–	Basic	–	–	NS
23^[29]^	M	17	SCAD	Uk	Neu, MR, CV	Car	–	Antipsy, Anticho	Yes	Yes	–	–	Vol	ND	–	Basic, CO_2_, BT	–	–	NS
24^[30]^	F	8	GAT2	Uk	No	Diet	–	–	–	–	–	–	TIVA (p)	ND	–	Basic, CO_2_, BT, ABGA	–	–	NS
25^[31]^	M	56	GAT2	4 YA	MR	No	–	AD	Yes	–	–	–	TIVA	ND	–	Basic, ABGA, invasive	–	–	NS
26^[31]^	M	56	GAT2	4 YA	MR	No	–	AD	Yes	–	LBP	TAP	Vol	ND	–	–	–	–	NS
27^[32]^	F	17	MCAD	Uk	–	Car	–	Contra	–	Yes	Lido	Inf	Sed	No	No	Basic	–	–	NS
28^[33]^	M	8 Mo	VLCAD	Birth	–	Diet	–	–	Yes	–	RPV	Inf	TIVA	ND	–	Basic, CO_2_, BT, ABGA	–	–	NS
29^[34]^	M	9	VLCAD	8 YA	Neu, CV, GI	Diet	–	–	Yes	–	Lido, BPV	Peni	Sed	No	No	–	–	–	NS
30^[35]^	F	20	MCAD	5 MA	No	Car	Yes	–	–	Yes	BPV	NA	No	No	No	–	–	–	NS
31^[36]^	F	24	VLCAD	16 Mo	Neu	–	–	–	Yes	–	–	–	Vol	ND	–	Basic, Glu	–	–	NS
32^[37]^	F	37	VLCAD	6 YA	MR	–	–	–	Yes	–	–	–	Vol	ND	–	Basic, BT	–	–	NS

1CD = primary carnitine deficiency, 2CD = secondary carnitine deficiency, ABGA = arterial blood gas analysis, Anticho = anticholinergic, Anticon = anticonvulsant, AD = antidiabetes, Antipsy = antipsychotic, Anxio = anxiolytic, Basic = non-invasive blood pressure and/or electrocardiogram and/or pulse oximetry, Birth = at birth, BPV = bupivacaine, BR = bed ridden, BT = body temperature, Car = carnitine, CO_2_ = CO_2_ monitoring, Contra = contraceptive, CPR = cardiopulmonary resuscitation, CPT-I = carnitine palmitoyltransferase I, CPT-II = carnitine palmitoyltransferase II, CV = cardiovascular, DCT = defect of carnitine transport, Dep = depolarizing neuromuscular blocker, DFAO = defect of fatty acid β-oxidation, DFAO Hx = defect of fatty acid β-oxidation history, Diet = high carbohydrate or frequent eating, Dx. = diagnosis, F = female, FHx. = family history, GA = general anesthesia, GAT1 = glutaric aciduria type 1, GAT2 = glutaric aciduria type 2, GI = gastrointestinal, Glu = glucose monitoring, IA = isovaleric acidemia, ICU = intensive care unit, Inf = infiltration, Invasive = A-line, Swan Ganz, and transesophageal echocardiography, IVA = intravenous anesthesia, LA = local anesthetic, LE = lipid emulsion, Lido = lidocaine, LBP = levobupivacaine, M = male, MA = month ago, MCAD = medium-chain acyl-CoA dehydrogenase deficiency, MCCD = 3-methylcrotonyl-CoA carboxylase deficiency, MCT = medium-chain triglyceride, Mo = month, MPV = mepivacaine, MR = muscular or respiratory, Mx. = management, NA = neuraxial block, ND = non-depolarizing neuromuscular blocker, Neu = neurologic, NMB = neuromuscular blocker, NS = non-specific or uneventful, Peni = penile block, Ren = renal, RPV = ropivacaine, SCAD = short-chain acyl-CoA dehydrogenase deficiency, Sed = sedation, Severe = severe event requiring adrenaline or intensive care unit care, Sx = symptoms, T. of Dx. = timing of diagnosis, TAP = transversus abdominis plane block, This = this time, TIVA = total intravenous anesthesia, TIVA (P) = total intravenous anesthesia using propofol, Tx = treatment, Uk = unknown, Vol = volatile anesthetic, Vol (P) = volatile anesthesia involving induction with propofol, VLCAD = very long-chain acyl-CoA dehydrogenase deficiency, VPA = valproic acid, YA = year ago, – = not recorded.

The surgeries performed under general and regional anesthesia included delivery (N = 5; 15.62%), osteotomy, adenotonsillectomy (N = 2 each), poster fossa decompression, tracheostomy, dental rehabilitation, appendectomy, suction lipectomy, loop excision because of cervical dysplasia, the removal of pancreas tumor, language mapping under consciousness sedation, craniotomy, posterior spine fusion, orchiectomy, minor orthopedic surgery, ventricular septal defect closure, odontectomy, coronary artery bypass graft, laparoscopic cholecystectomy, tooth extraction, muscle biopsy and percutaneous gastrostomy, the resection of phimosis, the removal of neck cyst, and laparoscopic ovarian cystectomy (N = 1 each). Twenty-two patients (68.9%) were pre-operatively administered 10% or 5% dextrose to prevent hypoglycemia induced by pre-operative fasting (Table 1). L-carnitine was pre-operatively or regularly administered in 11 cases (34.37%), including defects in the fatty acid β-oxidation pathway (N = 6), secondary carnitine deficiency (N = 3; 3-methylcrotonyl-CoA carboxylase deficiency: N = 1, glutaryl-CoA dehydrogenase deficiency: N = 1, and isovaleryl CoA dehydrogenase deficiency: N = 1), and primary carnitine deficiency (N = 2) (Table 1). Local anesthetics, such as bupivacaine, ropivacaine, lidocaine, or mepivacaine were administered for regional or local anesthesia in 14 cases (43.75%), with or without general anesthesia (Table 1). While neuraxial block was performed in 7 cases (21.8%), local infiltration was performed in 5 cases (15.6%) (Table 1). Transversus abdominis plane block and penile block were performed in 1 case each (Table 1). Volatile anesthetics, including nitrous oxide, oxygen, halothane, enflurane, isoflurane, desflurane, and sevoflurane, were used in 17 cases (53.12%) (Table 1). Total intravenous anesthesia involving propofol-fentanyl, propofol-fentanyl-morphine, dexmedetomidine-midazolam-remifentanil, and remifentanil-midazolam was conducted to 6 cases (18.75%) (Table 1). Moreover, total intravenous anesthesia involving propofol was performed in 3 cases (9.37%) (Table 1). Non-depolarizing neuromuscular blockers, including cis-atracurium, atracurium, rocuronium, and vecuronium, were used in 17 cases (53.12%) that underwent general anesthesia using volatile and intravenous anesthetics (Table 1). However, succinylcholine was used in 2 cases (6.25%) involving patients with primary carnitine deficiency and CPT-I deficiency (Table 1). Intraoperative monitoring included blood pressure, electrocardiogram, end-tidal carbon dioxide concentration, body temperature, blood glucose, pulse oximeter, monitoring of neuromuscular blockade, and arterial blood gas analysis (Table 1). Unlike routine monitoring during anesthesia, intraoperative blood glucose monitoring was performed in 7 patients. However, this was not recorded in 12 cases.

Intraoperative and postoperative adverse events, including sudden cardiac arrest, sudden sinus bradycardia, ventricular arrhythmia, severe hypotension, and coma in the postanesthetic care unit, occurred in 4 cases (12.5%; Table 1). Of these cases, 2 patients had never been diagnosed with fatty acid β-oxidation disorder before the event and received only basic intraoperative monitoring and end-tidal carbon dioxide concentration during anesthesia. Treatment of this adverse event included cardiopulmonary resuscitation, epinephrine, ephedrine, atropine, carnitine, 20% glucose, lipid emulsion, and mechanical ventilation (Table 1). Of the 4 cases involving severe adverse events, 2 patients recovered, and the remaining 2 patients with primary or secondary carnitine deficiency died (Table 1). Lipid emulsion was used to treat intractable hemodynamic instability due to bupivacaine toxicity in secondary carnitine deficiency, leading to reduced plasma bupivacaine concentration (Table 1).^[7]^ It may be caused by increased susceptibility to bupivacaine toxicity in secondary carnitine deficiency due to valproic acid (Table 1).^[7]^ One case with primary carnitine deficiency produced rhabdomyolysis and cardiac arrest following general anesthesia involving nitrous oxide, oxygen, and enflurane (Table 1).^[11]^

## 3 Discussion

**Figure 2:**
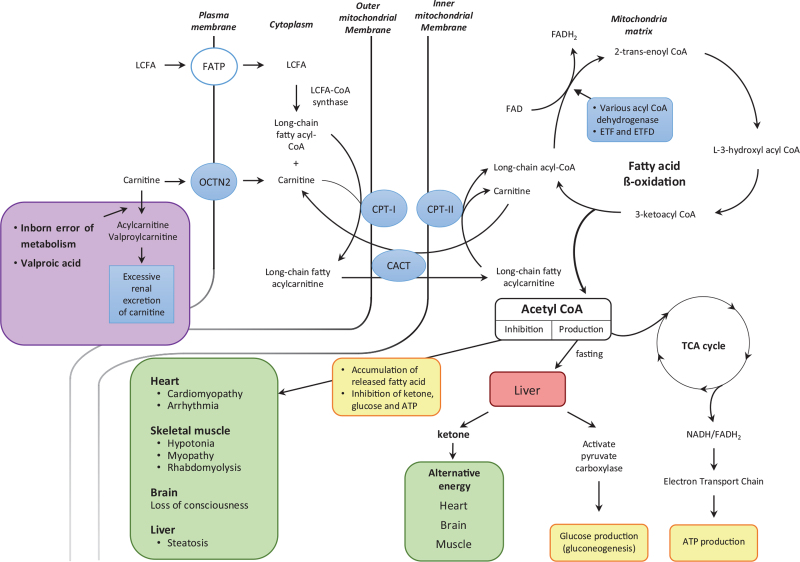
The pathway involving carnitine shuttle and subsequent fatty acid β-oxidation. Carnitine is transported to the cytoplasm via the organic carnitine transporter novel type 2 (OCTN2) in the plasma membrane.^[1]^ Long chain fatty acid (LCFA) is transported to the cytoplasm via the fatty acid transport protein (FATP).^[39]^ LCFA is converted to long chain fatty acyl CoA by long chain fatty acyl CoA (LCFA-CoA) synthase.^[39]^ Long chain fatty acylcarnitine is produced from long chain fatty acyl CoA and carnitine by carnitine palmitoyltransferase I (CPT-I) in the inner side of the outer mitochondria membrane.^[39]^ Long chain fatty acylcarnitine is transported from the cytoplasm to the intermembrane mitochondrial space, followed by the transport of long chain fatty acylcarnitine to the mitochondria matrix by carnitine acylcarnitine translocase (CACT).^[39]^ It gets split into long chain acyl CoA and carnitine by carnitine palmitoyltransferase II (CPT-II) in the mitochondria matrix.^[39]^ Carnitine is returned to the cytoplasm from the mitochondria matrix by CACT.^[39]^ Long chain acyl CoA undergoes fatty acid β-oxidation mediated by 4 enzymes, including acyl CoA dehydrogenase, enoyl CoA hydratase, hydroxyacyl CoA dehydrogenase, and keto acyl CoA thiolase.^[2]^ The first step of fatty acid β-oxidation is mediated by very long, medium, and short chain acyl CoA dehydrogenase, which involves electron transferring flavoprotein (ETF) and electron transferring flavoprotein dehydrogenase (ETFD).^[2]^ Acetyl CoA is one of the final products of fatty acid β-oxidation, which produces nicotinamide adenine dinucleotide hydrogen (NADH)/flavine adenine dinucleotide (FADH2) via the tricarboxylic acid (TCA) cycle, thus leading to adenosine triphosphate (ATP) production.^[2]^ In addition, acetyl CoA stimulates ketone and glucose production (via the activation of pyruvate carboxylase) in liver during fasting, thereby suggesting glucose sparing and glucose production, respectively.^[41]^ Ketone is produced by acetyl CoA during fasting in the liver and is used as alternative energy source in the skeletal muscle, heart, and brain during fasting, thus suggesting glucose sparing.^[41]^ However, the inhibition of acetyl CoA production caused by compromised fatty acid β-oxidation, which occurs because of carnitine deficiency (primary, secondary: because of an inborn error of metabolism or valproic acid) or the defect of carnitine transport (CPT-I or CPT-II deficiency) or fatty acid β-oxidation pathway (because of various acyl CoA dehydrogenase deficiency or a deficiency of ETF and ETFD), leads to cardiac arrhythmia and cardiomyopathy (heart), hypotonia and rhabdomyolysis (muscle), steatosis (liver), hypoglycemia, and the loss of consciousness (brain).^[^1^,^^2^^,^^39^^,^^41^^]^

Carnitine deficiency is divided into 3 types as follows: primary carnitine deficiency, secondary carnitine deficiency, and defects in carnitine transport. Primary carnitine deficiency is caused by genetic disorder (autosomal recessive) of the organic carnitine transporter novel type 2 in the plasma membrane (Fig. 2), which contributes to carnitine transport.^[1]^ Secondary carnitine deficiency is associated with enhanced renal excretion and inadequate dietary intake because of malabsorption, malnutrition, and drug treatments (e.g., valproic acid) (Fig. 2).^[1]^ In addition, very long and medium chain acyl CoA dehydrogenase deficiency is associated with defects in the fatty acid β-oxidation pathway, and may produce secondary carnitine deficiency via the accumulation of acylcarnitine, thus leading to the inhibition of renal carnitine absorption (Fig. 2).^[38]^ The defect in carnitine transport is attributed to the deficiency or inhibition of carnitine palmitoyltransferase (CPT) (-I and -II) and carnitine acylcarnitine translocase (CACT), which contribute to the carnitine shuttle involved in long-chain fatty acid transport to the mitochondrial matrix.^[39]^ Long chain fatty acids are transported to the cytoplasm through fatty acid transport proteins in the plasma membrane. Subsequently, long-chain fatty acyl CoA synthase produces long-chain fatty acyl CoA from long-chain fatty acids (Fig. 2).^[39]^ Long-chain fatty acylcarnitine, which is produced from long-chain fatty acyl CoA and carnitine by CPT-I, is transported from the cytoplasm to the intermembrane space of mitochondria (Fig. 2).^[39]^ Consequently, long-chain fatty acylcarnitine gets transported from the intermembrane space of mitochondria to the mitochondrial matrix by CACT (Fig. 2).^[39]^ Long-chain fatty acylcarnitine is split into long-chain fatty acyl CoA and carnitine by CPT-II in the mitochondrial matrix (Fig. 2), followed by the return of carnitine to the cytoplasm by CACT (Fig. 2).^[39]^ Long-chain fatty acyl CoA undergoes fatty acid β-oxidation to produce acetyl CoA, which subsequently produces nicotinamide adenine dinucleotide hydrogen and flavin adenine dinucleotide via the tricarboxylic acid cycle, thereby leading to adenosine triphosphate production via the electron transport chain (Fig. 2).^[2]^ The end stage of fatty acid β-oxidation produces acetyl CoA from long chain fatty acyl CoA, which is mediated by 4 enzymes—namely, acyl CoA dehydrogenase, enoyl CoA hydratase, hydroxyacyl CoA dehydrogenase, and ketoacyl CoA thiolase (Fig. 2).^[^2^,^^40^^]^ Thus, defects in the fatty acid β-oxidation pathway (N = 18), secondary carnitine deficiency (N = 5), primary carnitine deficiency (N = 4), and defects in carnitine transport (N = 4) inhibits fatty acid β-oxidation.^[2]^ In addition, ketone body (β-hydroxybutyrate, acetoacetate, and acetone) production by acetyl CoA generated by fatty acid β-oxidation in the liver during fasting is a predominant substitute for energy production in the heart, skeletal muscle, and brain (Fig. 2).^[41]^

The very long, medium, and short chain acyl CoA dehydrogenase (Fig. 2), which is dependent on the carbon number of fatty acids, is involved in the first step of fatty acid β-oxidation from long-chain fatty acyl CoA.^[2]^ Electron transferring flavoprotein and electron transferring flavoprotein dehydrogenase (Fig. 2), which contribute to the first step of fatty acid β-oxidation by very long, medium, and short chain acyl CoA dehydrogenase, are associated with the electron transport chain.^[^2^,^^40^^]^ Carnitine deficiency and defects in the downstream fatty acid β-oxidation pathway can affect the liver, heart, skeletal muscle, and brain by the accumulation of fat released from adipose tissues during fasting, which leads to steatosis (liver), cardiomyopathy (including arrhythmia), myopathy (including hypotonia and rhabdomyolysis), and the loss of consciousness (brain) because of hypoglycemia (Fig. 2).^[41]^ Furthermore, reduced acetyl CoA production caused by an inhibition of fatty acid β-oxidation during fasting prevents the production of ketones, which can be used as an alternative energy source (Fig. 2).^[41]^ This inhibition is an outcome of carnitine deficiency or defects in either carnitine transport or fatty acid β-oxidation pathway. In addition, acetyl CoA stimulates pyruvate carboxylase to activate gluconeogenesis in the liver, thus leading to the production of glucose (Fig. 2).^[41]^ Taken together, acetyl CoA production via fatty acid β-oxidation during fasting contributes to both glucose production via gluconeogenesis and glucose sparing via ketone production as an alternative energy source (Fig. 2).^[41]^ Thus, the inhibition of acetyl CoA production inhibits gluconeogenesis and subsequently produces hypoketotic hypoglycemia and the loss of consciousness in the brain (Fig. 2).^[41]^ Furthermore, considering the use of long-chain fatty acids as an energy source (energy: 80%) in the heart, carnitine deficiency and subsequent fatty acid β-oxidation inhibition lead to cardiomyopathy and myocardial depression by the accumulation of the released fatty acid and acylcarnitine.^[^2^,^^39^^,^^41^^]^ In addition, glutaric aciduria type II (N = 3; Table 1) because of the deficiency of electron transferring flavoprotein and electron transferring flavoprotein dehydrogenase produces fatty acid β-oxidation disorder.^[^40^,^^42^^]^ Bupivacaine reportedly inhibits CACT, which contributes to carnitine shuttle associated with long-chain fatty acid transport to the mitochondrial matrix.^[43]^

The pre-anesthetic laboratory examination in patients with carnitine deficiency or defects in the fatty acid β-oxidation pathway should include electrocardiogram, echocardiography, blood glucose, carnitine, creatine kinase, and serum transaminase (Table 1). Patients with primary carnitine deficiency have low levels of carnitine in the plasma (<5 μM/L), which necessitates life-long L-carnitine (100–200 mg/kg/day) treatment, which leads to the prevention of hypoglycemia and the improvement of cardiomyopathy and muscle weakness.^[^1^,^^39^^]^ However, considering the inefficacy of carnitine treatment in the defect of carnitine transport, carnitine treatment was not performed in such cases (N = 4; Table 1). Carnitine deficiency and defects in the fatty acid β-oxidation pathway necessitate hypoglycemia prevention by frequently eating and avoiding fasting. Therefore, glucose (5% and 10%) was pre-operatively used for the prevention of intraoperative hypoglycemia in 22 cases (68.75%; Table 1).^[1]^ Considering that fatty acids are major energy sources in catabolic states, such as fasting, vigorous exercise, infection, and surgery, the long-term goal of glucose supply, which prevents hypoglycemia in the acute stage, is to inhibit further fatty acid β-oxidation in patients with compromised fatty acid β-oxidation.^[41]^ This eventually results in the decreased release of fatty acids from adipose tissues.^[41]^ The pre-operative evaluation should focus on the following factors in patients with carnitine deficiency or defects in the fatty acid β-oxidation pathway (Table 1): the optimization of glucose level worsened by pre-operative fasting and stress, an evaluation of cardiac function, including echocardiography, and L-carnitine treatment (in carnitine deficiency). The pre-operative diagnosis of most patients (N = 28; 90.3%; Table 1) with fatty acid β-oxidation disorder warrants recording medical history. In addition, clinicians should document the family history (N = 6, 19.3%) associated with fatty acid β-oxidation disorder and other medication history (e.g., valproic acid), which can cause secondary carnitine deficiency and defects in fatty acid β-oxidation (Table 1).

Considering that propofol is dissolved in 10% intralipid containing 100% long-chain fatty acid, it should be avoided in patients with carnitine deficiency or defects in the fatty acid β-oxidation pathway. This is because propofol increases long-chain fatty acid loading in patients with compromised fatty acid β-oxidation.^[^25^,^^29^^]^ Thus, we surmised that only 6 (18.75%) of the 32 surgeries used propofol as an induction or maintenance agent of general anesthesia (Table 1).^[^7^,^^12^^,^^13^^,^^20^^,^^28^^,^^30^^]^ Furthermore, the probability of the propofol infusion syndrome increased upon the intravenous administration of propofol (>4 mg/kg/h) for more than 48 hours.^[44]^ The suggested underlying mechanism of propofol infusion syndrome includes the inhibition of CPT-I, fatty acid β-oxidation, and electron transport chain, similar to an acquired form of carnitine deficiency.^[^45^,^^46^^]^ Thus, it would be feasible to avoid propofol in patients with compromised fatty acid β-oxidation, notwithstanding small dosage and short infusion duration.^[^45^,^^46^^]^ However, total intravenous anesthesia using propofol was performed in 3 cases (9.37%; Table 1).^[^12^,^^20^^,^^30^^]^ One case underwent a bolus administration of 2 mg/kg propofol, followed by 8 mg/kg/h propofol for 3 hours.^[12]^ The remaining 2 cases involved continuous infusion (0.08 mg/kg/min and 0.016 mg/kg/min), which were supposedly small doses.^[^20^,^^30^^]^ In addition, total intravenous anesthesia or sedation, which used ketamine-fentanyl, remifentanil-dexmedetomidine-midazolam, or midazolam-alfentanil other than propofol, was performed in 4 patients with compromised fatty acid β-oxidation (Table 1).^[^24^,^^31^^,^^33^^,^^34^^]^ Considering CPT deficiency in some patients susceptible to malignant hyperthermia, the use of depolarizing muscle relaxant and inhalational anesthetic agent (sevoflurane, desflurane, and isoflurane) should be avoided in such patients.^[^47^,^^48^^]^ Dantrolene should be prepared to treat malignant hyperthermia. Thus, 3 patients of 4 patients with CPT-I or CPT-II received neuraxial block (Table 1). However, 1 patient with CPT-I deficiency underwent general anesthesia with a volatile anesthetic (sevoflurane) and depolarizing neuromuscular blocker (suxamethonium), and was admitted to the intensive care unit in coma status following adenoidectomy.^[17]^ At admission, she did not inform about being diagnosed with CPT-I deficiency; however, she recovered completely from coma after being treated with carnitine, dextrose, and oxygen.^[17]^ Regional anesthesia can be used to avoid malignant hyperthermia in patients with CPT deficiency. As the non-toxic dose of bupivacaine produces ventricular arrhythmia and hypotension in patients with secondary carnitine deficiency who exhibit plasma carnitine levels within or below the reference limits, a subtoxic dose of local anesthetic-induced cardiotoxicity should be considered cautiously.^[^6^,^^7^^,^^14^^]^ In addition, glucose infusion and intraoperative glucose monitoring should be maintained under regional anesthesia. Among all surgeries performed in patients with fatty acid β-oxidation disorder, deliveries accounted for the largest proportion (N = 5; 15.6%). Regional anesthesia, such as epidural anesthesia for delivery that includes careful cardiac monitoring, is reasonable in patients with CPT deficiency. Compared with the muscle carnitine levels before treatment, the levels were increased by the 6 months supply of L-carnitine in patients with infantile lipid myopathic carnitine deficiency; however, some patients did not reach the normal carnitine level in muscles.^[49]^ This necessitates an adequate amount of L-carnitine for a sufficient period to reach adequate levels in the skeletal muscle, heart, and liver. In addition, the plasma free carnitine level does not always reflect the tissue level.^[^6^,^^50^^]^ Intraoperative monitoring, which includes blood glucose, electrolyte, acid-base balance, creatine kinase, and cardiac monitoring, such as intraoperative echocardiography, is essential for patients with carnitine deficiency or defects in the fatty acid β-oxidation pathway. In particular, glucose is intravenously administered during the perioperative period, including fasting time. Moreover, glucose levels should be monitored intraoperatively. Secondary carnitine deficiency due to a deficiency of 3-methylcrontonyl-CoA carboxylase or isovaleryl-CoA dehydrogenase (Table 1), which is involved in leucine metabolism, increases the excretion of carnitine via carnitine conjugation (e.g., 3-hydroxyisovalreylcarnitine) (Fig. 2).^[^6^,^^13^^,^^51^^]^ Symptoms of secondary carnitine deficiency are mild, compared to primary carnitine deficiency.^[1]^ The prevention and treatment of hypoglycemia is important in patients with defects in the fatty acid β-oxidation pathway, such as very long and medium chain acyl CoA dehydrogenase deficiency. However, L-carnitine supplementation can be attempted in very long and medium chain acyl CoA dehydrogenase deficiency after low plasma carnitine is confirmed.^[^52^,^^53^^]^ Thus, carnitine supply observed in 6 patients with defects in the fatty acid β-oxidation pathway (N = 6; 19.3%; Table 1) may be associated with the treatment of secondary carnitine deficiency. Clinically relevant concentrations of inhalation anesthetics (halothane, isoflurane, and sevoflurane) slightly inhibit complex I of the electron transport chain.^[54]^ In addition, enflurane increases the fatty acid plasma concentration.^[55]^ Hence, the use of intravenous anesthesia with opioids, benzodiazepines, dexmedetomidine, etomidate, and non-depolarizing muscle relaxants would be reasonable in patients with compromised fatty acid β-oxidation.

## 4 Conclusions

In summary, frequent eating, short duration of fasting, and stress reduction (infection and fever) are essential to avoid hypoglycemia. Pre-operative evaluation should include plasma glucose and carnitine levels to confirm whether they are within the reference limits. Dextrose (5% and 10%) is intravenously administered during the pre-operative period, and glucose should be monitored pre-operatively, intraoperatively, and postoperatively. Moreover, general anesthesia using inhalation anesthetics and succinylcholine should be avoided in patients with CPT deficiency to prevent the occurrence of malignant hyperthermia. A subtoxic dose of local anesthetic may cause cardiotoxicity because of increased susceptibility to local anesthetic systemic toxicity in patients with carnitine deficiency. This warrants meticulous monitoring, including cardiac monitoring, during regional anesthesia in patients with compromised fatty acid β-oxidation. Moreover, propofol infusion should be avoided because propofol may not only induce propofol infusion syndrome but also increase long-chain fatty acid loading in patients with compromised fatty acid β-oxidation. Intravenous anesthesia using opioids (fentanyl and remifentanil), benzodiazepine (midazolam), etomidate, and dexmedetomidine would be more suitable for patients with compromised fatty acid β-oxidation.

## Author contributions

**Conceptualization:** Ho Kyung Yu, Ju-Tae Sohn.

**Data curation:** Ho Kyung Yu, Sunmin Kim.

**Formal analysis:** Seong-Ho Ok, Ju-Tae Sohn.

**Funding acquisition:** Seong-Ho Ok, Ju-Tae Sohn.

**Investigation:** Ho Kyung Yu, Seong-Ho Ok.

**Methodology:** Seong-Ho Ok, Ju-Tae Sohn.

**Supervision:** Ju-Tae Sohn.

**Validation:** Ho Kyung Yu, Seong-Ho Ok, Ju-Tae Sohn.

**Visualization:** Sunmin Kim.

**Writing – original draft:** Ju-Tae Sohn.

**Writing – review & editing:** Ho Kyung Yu, Seong-Ho Ok, Sunmin Kim, Ju-Tae Sohn.
